# What factors affect evidence-informed policymaking in public health? Protocol for a systematic review of qualitative evidence using thematic synthesis

**DOI:** 10.1186/s13643-016-0240-6

**Published:** 2016-04-14

**Authors:** Ben Verboom, Paul Montgomery, Sara Bennett

**Affiliations:** Centre for Evidence-Based Intervention, Department of Social Policy and Intervention, University of Oxford, Barnett House, 32 Wellington Square, Oxford, OX1 3DW, UK; Department of International Health, Johns Hopkins Bloomberg School of Public Health, 615 North Wolfe Street, Baltimore, 21205, MD USA

**Keywords:** Evidence-informed policy, Evidence-to-policy processes, Evidence use, Public health, Public health policy, Qualitative evidence synthesis, Qualitative research

## Abstract

**Background:**

Claims of and calls for evidence-informed policymaking pervade public health journals and the literature of governments and global health agencies, yet our knowledge of the arrangements most conducive to the appropriate use of evidence is incomplete and fragmented. Designing interventions to encourage evidence use by policymakers requires an understanding of the processes through which officials access, assess and use research, including technical and political factors related to evidence uptake, and the ways in which the policymaking context can affect these processes. This review aims to systematically locate, synthesise and interpret the existing qualitative work on the process of evidence use in public health policymaking, with the aim of producing an empirically derived taxonomy of factors affecting evidence use.

**Methods/design:**

This review will include primary qualitative studies that examined the use of research evidence by policymakers to inform decisions about public health. To locate studies, we will search nine bibliographic databases, hand-search nine public health and policy journals and scan the websites of relevant organisations and the reference lists of previous reviews of evidence use in policymaking. Two reviewers will independently screen studies, apply inclusion criteria and appraise the quality of included studies. Data will be coded inductively and analysed using thematic synthesis. An augmented version of the CASP Qualitative Checklist will be used to appraise included studies, and the CERQual tool will be used to assess confidence in the review’s findings. The review’s results will be presented narratively and in tabular form. Synthesis findings will be summarised as a taxonomy of factors affecting evidence use in public health policymaking. A conceptual framework explaining the relationships between key factors will be proposed. Implications and recommendations for policy, practice and future research will be discussed.

**Discussion:**

This review will be the most comprehensive to date to synthesise the qualitative literature on evidence use by public health policymakers and will be the first to apply a formal method of qualitative metasynthesis to this body of evidence. Its results will be useful both to scholars of evidence use and knowledge translation and to decision-makers and academics attempting to influence public health policy.

**Electronic supplementary material:**

The online version of this article (doi:10.1186/s13643-016-0240-6) contains supplementary material, which is available to authorized users.

## Background

The recent popularisation of evidence-based medicine, which calls for the explicit, judicious and conscientious use of up-to-date research evidence in clinical decision-making, has prompted discussion about the role of research evidence in informing policy-level decision-making, leading to calls for more systematic and appropriate evidence use by actors involved in crafting public health policy.

Designing interventions to encourage the appropriate use of evidence by public health policymakers requires an understanding of the processes through which bureaucrats and politicians access, assess and use evidence, including the technical factors (i.e. barriers and facilitators) related to evidence uptake. It also requires an appreciation for the ideological nature of policymaking in general [1], and (public) health policymaking in particular [2], and the processes through which political factors can affect whether and how evidence translates into policy [3]. Policy decisions related to the health of populations, whether at the subnational, national or international level, are likely to be particularly controversial and politically charged. The ongoing debate about the extent to which the state should be engaged in promoting public health and access to health services [4, 5] and the often unequal distribution of the benefits of population-level health interventions [6] are just two reasons for this.

Policymaking inevitably involves (often controversial) trade-offs between different values, priorities, and interests, including considerations related to public opinion, expected health outcomes, intervention cost, perceived fairness and equity and ethics [7]. An understanding of how decision-makers weigh up research evidence against these competing priorities is a prerequisite to developing strategies to encourage these actors to use evidence more appropriately.

A growing body of primary qualitative literature has examined the role of research evidence in public health decision-making [8]. However, previous efforts to systematically review this work [8–10] have primarily taken an ‘aggregative’ or ‘synoptic’ approach, that is, they have pooled and summarised data from existing primary studies without reinterpreting reported findings across studies to generate novel theoretical insights. The evolving family of qualitative review methods collectively termed ‘qualitative evidence synthesis’ or ‘qualitative metasynthesis’ [11] is defined by the production of ‘higher-level’ (sometimes called ‘third-order’ [12]) theoretical constructs that ‘go beyond’ providing an aggregation of individual study findings to more comprehensively explain phenomena of interest. Importantly, qualitative metasynthesis involves integrating findings from multiple qualitative studies and, through considering the body of included study reports as a whole, producing inferences and interpretations that are not located in or derivable from any one included research report [11].

In this review, we will apply the metasynthetic technique of thematic synthesis [13] to analyse and interpret the existing qualitative work on the process of evidence use in public health policymaking, with the aim of producing an empirically derived taxonomy of factors affecting this process. The specific objectives of this review are:

1. To locate and synthesise all qualitative evidence on the use of research evidence in public health policymaking, including data on:
  Factors (e.g. barriers and facilitators) associated with the (appropriate) use of evidence
  The process(es) through which evidence is accessed, valuated (i.e. compared to competing inputs) and applied to policymaking
  Policymaker perceptions of the (appropriate) role of research evidence in public health policymaking in general and their work in particular
2. Using the data obtained through the first objective, to produce a taxonomy of the factors impacting upon evidence-informed policymaking in public health
3. To preliminarily identify, through the examination of subgroups of included studies, factors affecting evidence use that may be dependent on the context in which the activity of policymaking takes place

## Methods/design

We explored the possibility of registering this protocol in the PROSPERO database, but learned that (as of early-2016) PROSPERO only accepts systematic reviews with a ‘health-related outcome’. As our review does not meet this criterion, it has not been registered in PROSPERO. However, we hope that the a priori publication of this protocol serves some of the same purposes as registration, namely increasing transparency and reducing the likelihood of bias and unnecessary duplication of efforts [14].

### Criteria for considering studies for this review

In this section, we outline the criteria against which studies will be included or excluded from the review. Briefly, in order to be included in the review, a study must:

- Be a qualitative study, published or unpublished
- Examine *policy* activities, processes and/or decisions in *policy* settings
- Report data concerning the use of *research evidence* to inform decisions about *public health*

In the subsections that follow, we provide a more detailed explanation of and rationale for these inclusion criteria.

#### Types of studies

This review will only include qualitative studies that report primary data. For the purposes of this review, we will use the following definition of ‘qualitative study’: a study that uses qualitative methods both for data collection and data analysis. This definition is consistent with that used in several recent qualitative syntheses [15–17] and was cited as one useful definition in the Cochrane Qualitative and Implementation Methods Group supplementary guidance on qualitative evidence synthesis [18]. Methods of qualitative *data collection* include (but are not limited to) interviews, focus groups and participant observation methods, while methods of qualitative *data analysis* include, for example, thematic analysis, content analysis, discourse analysis and grounded theory approaches. This definition of qualitative study excludes, for example, studies in which data were collected through interviews or focus groups, but were analysed exclusively through quantitative methods (e.g. tallies, descriptive statistics, etc.). We will include mixed methods studies that used both qualitative and quantitative methods, provided that it is possible extract data derived only from the qualitative methods.

We will not exclude studies according to the epistemological assumption(s) and/or theoretical tradition(s) on which they were based. That is, we will include all work within the broad ‘qualitative paradigm’.

Studies will not be excluded from the review on the basis of any hierarchy of qualitative evidence or criteria related to study quality. However, we recognise the possibility that including all eligible qualitative studies regardless of methodological quality may risk biasing the review’s findings [19]. As described below, a quality appraisal tool will be applied to all included studies prior to data analysis. Using sensitivity analysis (also discussed below), we will assess the potential undue influence of low scoring studies on our findings [20].

Studies will not be excluded on the basis of publication status (e.g. type of publication), publication date or the language in which the study was reported.

#### Types of settings and participants

This review will include studies reporting on policy activities, decisions, and/or processes with an explicit (though not necessarily exclusive) focus on public health issues. Studies reporting data derived from human participants (i.e. via interviews, focus groups or observation, as opposed to documents) will be included in the review if their participants included policymakers engaged in such activities. There is no universally accepted definition of the population ‘policymakers’ [21, 22]. For the purposes of this review, the population ‘policymakers’ includes government officials of any rank who are either elected (i.e. politicians) or appointed (i.e. civil servants, policy advisors and/or bureaucrats), working at the sub-national (e.g. local, state, provincial), national or international/global (i.e. intergovernmental) levels. Consistent with previous reviews [23, 24], we distinguish policymakers from managers (i.e. program managers, healthcare executives, management consultants, with supervisory and management responsibilities in healthcare and public health organisations) and service providers (i.e. front-line practitioners who typically make decisions about individual patient care, including physicians and nurses). Studies that exclusively include managers and/or service providers will be excluded.

If there is any lack of clarity within study reports about the role(s) of study participants and, as a consequence, uncertainty about whether they fit this definition of ‘policymakers’, we will contact original study authors for clarification.

Unlike in previous reviews of evidence use in public health [10] and non-health sectors of policy [25], studies will not be excluded on the basis of the country in which the study was conducted. The rationale for this inclusiveness is to capture the potential influence on evidence use of factors related to the political, economic and social context in which policymaking activities take place.

#### Subject matter of studies

To be eligible for inclusion, studies must explore the use or reported use of some type of *research evidence* in policy processes and/or decisions related at least in part to *public health*.

For the purposes of this review, public health policy decisions are those taken with the explicit goal of promoting the health of the population (whether at the sub-national, national or international level). This excludes policy decisions related to the provision of individual-level clinical interventions, unless these have an explicitly stated public health goal (as in the case of most immunisation policies). However, recognising the now widely appreciated importance of the social determinants of health [26] and understanding that policy decisions made outside of ministries and departments of public health, across a variety of sectors (e.g. transport, housing, crime), can have meaningful impacts on health [27], studies of policymakers with non-health portfolios will be included if population health, or the relationship between their decisions and public health outcome(s), is an explicit focus of the research.

For this review, research evidence will be defined as research produced by academic researchers and/or published in academic journals. This definition is similar to that used in a recent systematic review of evidence use in non-health settings [25], whose authors found that their original attempt to use a broader definition of research evidence produced results so conceptually heterogeneous that a meaningful synthesis was unfeasible. Included studies may examine the use of research evidence in general, or a specific category or ‘form’ of research evidence, including, for example, reports of randomised controlled trials, systematic reviews and/or meta-analyses and evidence summaries or overviews of reviews.

### Search methods for identification of studies

We plan to electronically search the following bibliographic databases without restriction by date:

- Applied Social Sciences Index and Abstracts (ASSIA)
- Conference Proceedings Citation Index—Social Science and Humanities
- EMBASE
- Global Health
- International Bibliography of the Social Sciences (IBSS)
- MEDLINE
- SCOPUS
- Social Sciences Citation Index (SSCI)
- Worldwide Political Science Abstracts (WPSA)

Search strategies for each database have been designed in consultation with an information retrieval specialist. In general, the strategies prioritise sensitivity in order to capture all studies relevant to the research question without restriction on the basis of language, location or publication status of the study. Where possible and appropriate, we plan to use a qualitative study design filter in order to enhance specificity [28, 29]. See Additional file 1 for our MEDLINE search strategy.

We also plan to hand-search the following journals (or specific sections thereof, where indicated) published since the beginning of 2010:

- *BMC Public Health* (Sections: Global health; Health policies, systems and management in high-income countries; Health policies, systems and management in low and middle-income countries)
- *Evidence & Policy*
- *Health Policy*
- *Health Policy & Planning*
- *Health Research Policy and Systems*
- *Implementation Science*
- *Journal of Health Politics, Policy & Law*
- *Milbank Quarterly*
- *Social Science and Medicine*

To supplement these sources, we will scan the ‘publications’ and/or ‘documents’ sections of the websites of relevant foundations, agencies and organisations (e.g. Evidence to Policy Initiative (E2Pi), University of California at San Francisco) and the reference lists of our included studies and of previous reviews of evidence use in policymaking [3, 8–10, 25]. As needed, experts and authors of included studies will be contacted to access unpublished data of studies located through the above methods and to obtain information about any as yet unpublished studies.

### Data collection and analysis

In this section, we describe the planned methods for selecting studies, extracting and managing data, assessing the quality of included studies and confidence in the review findings and analysing and presenting the review findings.

#### Selection of studies

Study screening and selection will be conducted according to standard systematic review methods [30] using EndNote X7 software (Thomson Reuters). Two reviewers will independently screen all titles and abstracts. Full-text versions of records deemed potentially relevant by at least one reviewer will be obtained for further review. Both reviewers will then independently screen the full-text versions of all potentially relevant articles for inclusion in the review. Any disagreements will be resolved through conference and, if necessary, deferral to a third reviewer. Reasons for the exclusion of studies at the full-text review stage will be recorded, tabulated and provided in an appendix to the final review article.

#### Data extraction and management

There is no universally accepted approach to extracting data for the purposes of qualitative metasynthesis. Strategies vary from the very selective to the very inclusive. In extremely inclusive approaches, the entire texts of included papers are essentially treated as data, while in more selective approaches, findings are only extracted from included studies when they are explicitly supported by direct quotations from study participants in the text of the article [18]. This latter approach risks missing findings that on the individual study level are perhaps of secondary importance, but collectively may emerge as important recurrent themes across studies. We will therefore adopt an approach emphasising inclusiveness, since this review is interested in the full breadth of factors affecting evidence use in public health policy.

Since the ‘informants’ in qualitative reviews are the original study authors (not the studies’ participants), all author interpretations of study results (in the form of themes, categories, diagrams, tables, etc.) qualify as data for this review [15, 18]. Adopting a version of Thomas and Harden’s [13] approach, we will extract all data labelled by study authors as results/findings, etc. and discussion/conclusion(s)/interpretation(s), etc. Data will be extracted verbatim from study papers directly into NVivo-11 software (QSR International).

In addition to extracting results and discussion sections into NVivo, the following information will be recorded for each included study:

- Basic study information (authors, title, year(s) of data collection, year of publication)
- Brief summary of the study’s focus, phenomena of interest, and theoretical/philosophical basis
- Study design (including sampling procedures) and description of qualitative methods used (e.g. interviews, observation, document analysis)
- Description of the study setting, policymaking context, level of policymaking (i.e. subnational, national or international/global) and country or countries in which the activity of policymaking took place (as well as the country’s income level *at the time of data collection* according to the World Bank country classification system)
- Description of participants (type of policymaker, rank/title, gender, etc.)

BV will extract these data from all included studies using a tabular data extraction form in Microsoft Excel; a second reviewer will assess the extracted data for accuracy against original study reports. Discordant interpretations will be discussed and resolved. Persistent disagreements will be resolved in consultation with PM. These descriptive study-level data will be reported in a summary table included in the final report and will be used in exploratory sub-group analysis (discussed below).

#### Assessment of study quality

Study quality in this review will be assessed according to an adapted version of the Critical Appraisal Skills Programme’s (CASP) tool for appraising qualitative research [31]. CASP is arguably the most ‘user-friendly’ of the widely used tools; however, in a comparative assessment of three popular critical appraisal tools, CASP was found wanting in terms of sensitivity to descriptive, interpretive and theoretical validity [32]. To address these limitations, we have augmented the CASP tool with four items adapted from the Joanna Briggs Institute Qualitative Assessment and Review Instrument [33]. The adapted CASP instrument (Additional file 2) contains 12 items, all of which can be answered with either ‘Yes’, ‘No’, or ‘Unclear’. For the purpose of aiding interpretation of the review findings, included studies will be assigned an overall score of ‘high’, ‘moderate’ or ‘low’ overall methodological quality according to a holistic reading of included papers, guided by consideration of these 12 items. It is important to note that these items are not designed to yield an overall numerical ‘score’ for the methodological quality of studies. Rather, the questions are designed as prompts to guide the reviewers in a critical reading of the studies. Indeed, as there is no consensus on the relative weight that should be ascribed to any individual characteristic of study quality, the presentation of a simple summed score of the tool’s items would risk being more misleading than informative.

BV and a second reviewer will independently pilot the adapted CASP instrument on a random sample of five included studies. The results of the two reviewers’ assessments will be compared and any discordant interpretations of items will be discussed with the broader review team to establish a consensus moving forward. Following the pilot stage, BV and the second reviewer will independently assess the quality of all included studies. Disagreements will be discussed and resolved. Persistent disagreements will be resolved by consulting PM. Quality ratings assigned to each study, and the rationale for these decisions (including any disputes and how they were resolved), will be explicitly detailed in an appendix to the final report.

As mentioned above, the results of the quality assessment will not be used to exclude studies from the review, but quality ratings will be used to inform interpretation of the data [34] and will be used in concert with the Confidence in the Evidence from Reviews of Qualitative Research (CERQual) tool to assess the confidence in the review’s findings (discussed below).

As well, post hoc sensitivity analysis will be undertaken to assess the potential influence of study quality on the review’s findings. This approach is analogous to the sensitivity analyses commonly conducted in quantitative reviews [20, 35]. We will estimate the robustness of the review’s findings to the inclusion/exclusion of studies receiving a ‘low quality’ rating to test for the potential undue influence of severely methodologically flawed studies [36].

#### Data synthesis

Data will be analysed using NVivo-11 software according to Thomas and Harden’s [13] method of qualitative thematic synthesis. Thematic synthesis involves the line-by-line coding of the text of included studies to produce so-called descriptive themes within studies, followed by the re-interpretation and synthesis of these newly organised data across studies to produce higher-order ‘analytical themes’ that ‘go beyond’ the findings of the individual primary studies. This potential generation of new theory via the production of higher order themes represents a synthesis step hitherto not attempted in reviews of evidence use in public health policymaking. No themes will be specified a priori to guide the review. Rather, themes will be allowed to emerge inductively as we interpret individual study data and synthesise data across studies.

The synthesis procedure will follow the three stages of thematic synthesis described by Thomas and Harden [13], the first two of which take place concurrently: (1) coding text, (2) developing descriptive themes and (3) developing analytical themes.

During the first and second stages, the data from each included study will be considered in isolation. BV will read and re-read the text of each study in NVivo-11 and will develop codes to describe the meaning and content of the text line by line. Sections of text (e.g. sentences) may be assigned a single code or multiple codes. As codes emerge and accumulate during this process, BV will organise them hierarchically, as appropriate, to develop descriptive themes to explain the data. During this stage, all of the text assigned to each code will periodically be checked for interpretive consistency to determine whether new codes are necessary and to contemplate whether some codes should be collapsed. Throughout this process, other team members will be consulted regularly to discuss coding decisions and the validity of the emerging list of descriptive themes, and amendments will be made as necessary.

During the third stage of thematic synthesis, ‘higher order’ analytical themes are developed from critical examination of the aggregated descriptive themes developed in stage two [13]. At this point in the analysis, BV will reconsider the set of data, now coded across studies and organised into descriptive themes, and interrogate it for newly emerging cross-study themes. As in stage two, other team members will be consulted regularly to discuss the appropriateness of decisions related to the assignment of analytical themes. Following consultations, amendments to themes will be made as necessary, and the data will be revisited and considered in the context of any newly emergent themes. This iterative process will continue until examination of the data ceases to yield new analytical themes.

As the synthesis process progresses, interpretations of the emerging themes will be summarised as a taxonomy of ‘factors’ affecting evidence use in public health policymaking, categorised according to the analytical themes. As new findings emerge from the interrogation of the data, the list of factors will be amended as appropriate.

An auditable ‘decision trail’ tracking the supporting text for each theme, and the study/ies from which this text was drawn, will be recorded in NVivo throughout the process of data synthesis. Tracking (and transparently reporting) the studies that contribute to each ‘finding’ of the review will facilitate confidence assessments and subgroup analysis (both discussed below) and will enhance the ease with which the review can be critiqued and reproduced.

#### Assessment of confidence in review findings

The CERQual approach will be used to provide an assessment of the level of confidence (certainty) in each of the review’s findings [37]. In the context of qualitative evidence synthesis, ‘confidence’ refers to the extent to which a review finding can be considered a reasonable representation of the phenomenon of interest; for example, an assessment of high confidence would signify that the phenomenon of interest is unlikely to differ substantially from the review finding. The CERQual tool, which is currently under development, draws on the principles used to develop the Grading of Recommendations, Assessment, Development and Evaluations (GRADE) approach [38]. It emerged in 2010 to assist a World Health Organization guideline development panel in using synthesised qualitative evidence to inform their recommendations. At least six other reviews [15, 39–43] have utilised the CERQual approach or an earlier version of it.

Confidence is assessed at the level of the individual ‘review finding’; that is, through the process described below, each ‘finding’ from the synthesis will be assigned a confidence rating. The current version of the CERQual tool assesses confidence in the evidence on the basis of (1) the *methodological limitations* of the studies that contributed to each review finding, (2) the *relevance* of the included studies to the review question, (3) the *coherence* of each review finding and (4) the *adequacy* of the data contributing to each review finding. The *methodological limitations* of included studies refer to the extent to which there are problems in the design or conduct of the primary studies that contributed evidence to a review finding. The *relevance* of the included studies to the review question refers to the extent to which the review finding is applicable to the context (i.e., the population, setting and phenomenon) in which the review is interested. The *coherence* of a review finding refers to the extent to which the ‘pattern’ that constitutes a review finding is consistent across multiple individual studies (or, alternatively, incorporates explanations for variations in the finding across individual studies). The *adequacy* of data refers to the richness (and ‘thickness’) and quantity of data supporting a given review finding.

CERQual requires that review authors form what are ultimately subjective judgements based on criteria that are somewhat open to interpretation: there are no straightforward, algorithmic criteria upon which to rate studies in these four domains. For the purposes of this review, assessments will be made as follows:

1. An assessment of the *methodological limitations* of each review finding will be made on the basis of the quality ratings assigned to individual studies that contributed to that review finding (using the adapted CASP tool; described above). That is, we will take into consideration the assessed quality of all studies tied to a given review finding and decide whether or not each review finding was *generally* drawn from well-conducted studies.
2. The data contributing to a review finding will be deemed highly *relevant* when the contexts and phenomena of the primary studies underlying a review finding are not substantively different from the context and phenomena of interest in the review. Relevant studies will be those explicitly and centrally focused on research evidence use by public health policymakers. Studies in which the use of evidence is considered, but is peripheral to their central focus, will be considered less relevant. Similarly, studies in which public health is of secondary concern to the policymakers will also be deemed less relevant.
3. A review finding will be considered highly *coherent* when it is manifested across multiple contexts (i.e. studies) in a consistent pattern or where any significant variation in the finding across studies is plausibly explained by the finding itself (i.e. the finding is coherently context-dependent). Findings that are inconsistent and/or contradictory across studies, and where contradictions and inconsistencies are unexplained, will be deemed less coherent.
4. The *adequacy* of the data contributing to a finding will be determined by assessing the number of studies contributing to a finding, alongside the perceived ‘richness’ and ‘thickness’ of the data reported in those studies. A review finding that is supported by several primary studies of moderate richness/thickness and/or a few primary studies of high richness/thickness will be rated as being supported by adequate data. A finding that is supported by only one or few primary studies of moderate or low richness/thickness, or by any number of studies with only low richness/thickness, will be rated as being supported by less adequate data.

After assessing each of the four components, we will make a judgement about the overall confidence in each review finding. Findings will be assessed as having ‘high’, ‘moderate’, ‘low’ or ‘very low’ confidence. The developers of CERQual define these four levels of confidence as follows:

- High confidence: It is *highly likely* that the review finding is a reasonable representation of the phenomenon of interest
- Moderate confidence: It is *likely* that the review finding is a reasonable representation of the phenomenon of interest
- Low confidence: It is *possible* that the review finding is a reasonable representation of the phenomenon of interest
- Very low confidence: It is *not clear* whether the review finding is a reasonable representation of the phenomenon of interest

BV and a second reviewer will independently apply the CERQual tool to the review findings. The results of both assessments will be compared and discordant assessments discussed until consensus is achieved for each finding. Any persistent disagreements on confidence ratings will be resolved in consultation with PM. The final results of the CERQual assessment will be reported in tabular form (discussed below) and will be incorporated into the narrative explanation of review’s results.

#### Subgroup analysis

As appropriate, we will perform subgroup analysis to explore possible variation in the review’s findings according to three categories of study-level characteristic:

- Type of qualitative method(s) used (e.g. interviews/focus groups versus observation versus document analysis)
- Policy context (i.e. subnational versus national versus international/global)
- Economic context according to World Bank country classifications (i.e. studies conducted in high-income countries versus those conducted in either low-, lower-middle-, or higher-middle-income countries)

Examining differences in findings according to the qualitative methods used in included studies may help to inform the design of future work in this area. For instance, it is possible that more political and/or less socially desirable characteristics of evidence use or non-use are more difficult to capture using methods of self-report (i.e. individual or group interviews) than through analysis of policy documents or participant observation methods. The content of our findings derived from each of these methods may shed light on such discrepancies.

Exploring differences in findings across contexts could improve our understanding of the nature of public health evidence use at different governance levels and in different economic contexts and could help to inform the specification of interventions to encourage evidence-informed policymaking for different types of policymakers. These results may be also used to generate hypotheses about context-specific evidence use for future quantitative studies in this area. For example, these results could inform subgroup analyses in future reviews of the effectiveness of knowledge translation interventions for policymakers.

#### Presentation of findings

The study design features, participant and setting characteristics, data collection and analysis methods and reported findings of all included studies will be summarised in tabular form and described narratively.

Results of the thematic synthesis and of the CERQual assessments will be tabulated in a ‘summary of qualitative findings table’. The review findings (factors affecting evidence use) will be classified into categories, informed by the analysis and listed in a table. A brief description of each factor will be provided, along with each factor’s ‘confidence’ rating. This exercise will produce an empirically derived taxonomy of factors related to evidence use in public health policy that can be interrogated and further refined in future empirical work (both qualitative and quantitative). The review’s findings will also be summarised visually in a proposed conceptual framework explaining the relationship between the key factors affecting evidence use.

#### Reporting methods

This protocol was developed and reported according to the Preferred Reporting Items for Systematic Reviews and Meta-analyses (PRISMA)-P Statement for reporting systematic review protocols [44]. See Additional file 3 for a completed PRISMA-P checklist. The review’s methods and results will be reported according to the ENTREQ (enhancing transparency in reporting the synthesis of qualitative research) statement for reporting syntheses of qualitative studies [45]. The final literature searches employed will be reported using the STARLITE (sampling strategy, type of study, approaches, range of years, limits, inclusion and exclusions, terms used, electronic sources) standards for reporting literature searches [46], and search and screening results will be summarised and presented using the flowchart described in the PRISMA statement [47]. Any deviations from this protocol will be recorded by BV and reported in the final review report.

## Discussion

Claims of and calls for evidence-informed policymaking pervade public health journals and the literature of governments and international agencies, yet our knowledge of the conditions and arrangements most conducive to the appropriate use of evidence is incomplete and fragmented. While much primary research has been conducted to examine the factors affecting evidence use by public health policymakers [8], this literature remains largely unsynthesised. This review will be the most comprehensive to date to synthesise the qualitative literature on evidence use by public health policymakers and will be the first to apply a formal method of qualitative metasynthesis to this body of evidence. Its results will be useful both to scholars of evidence use and knowledge translation and to decision-makers and academics attempting to influence public health policy.

## Additional files

Additional file 1: Sample search strategy - Strategy used to conduct search in MEDLINE database (via OVID). (PDF 28 kb) Additional file 2: Adapted CASP form - Tool that will be used to assess the methodological quality of included studies. (PDF 27 kb) Additional file 3: PRISMA-P Checklist - Completed checklist demonstrating adherence to PRISMA-P guidelines for reporting systematic review protocols. (PDF 121 kb)

## Abbreviations

CASP: Critical Appraisal Skills Programme
CERQual: Confidence in the Evidence from Reviews of Qualitative Research
E2Pi: Evidence to Policy Initiative
ENTREQ: enhancing transparency in reporting the synthesis of qualitative research
GRADE: Grading of Recommendations, Assessment, Development and Evaluations
PRISMA: Preferred Reporting Items for Systematic Reviews and Meta-analyses
STARLITE: sampling strategy, type of study, approaches, range of years, limits, inclusion and exclusions, terms used, electronic sources
