# Supplementary material for: What factors affect evidence-informed policymaking in public health? Protocol for a systematic review of qualitative evidence using thematic synthesis
Source: Syst Rev. 2016 Apr 14;5:61. doi: 10.1186/s13643-016-0240-6 (PMC4831125; doi:10.1186/s13643-016-0240-6)
Supplement: Additional file 1: — Sample search strategy - Strategy used to conduct search in MEDLINE database (via OVID). (PDF 28 kb) [file 13643_2016_240_MOESM1_ESM.pdf]

Sample search strategy – MEDLINE (OVID)

1	exp Evidence-Based Practice/ or exp Knowledge Management/ or ("evidence based" or "evidence informed" or "evidence use" or "knowledge translation" or "knowledge transfer" or "knowledge exchange" or "knowledge broker*" or "knowledge mobilization" or "knowledge mobilisation" or "knowledge use" or "research use" or (evidence* adj2 (utilis* or utiliz* or uptake* or diffus* or disseminat*)) or (research* adj2 (utilis* or utiliz* or uptake* or diffus* or disseminat*)) or (knowledge* adj2 (utilis* or utiliz* or uptake* or diffus* or disseminat*))).tw.
2	exp Policy/ or exp Public Policy/ or exp Health Policy/ or exp Policy Making/ or exp Decision Making/ or exp Administrative Personnel/ or (policy* or policies or decision*).tw.
3	exp Public Health/ or exp Preventive Medicine/ or exp Health Promotion/ or exp Social Medicine/ or exp Health Planning/ or exp Regional Health Planning/ or exp Community Health Planning/ or exp Community Health Services/ or ("public health" or "population health" or (population* and health) or "preventive medicine" or prevent* or "health promotion" or "promotion of health" or "social medicine" or "health planning" or "community health").tw.
4	1 and 2 and 3
5	exp qualitative research/
6	(qualitative or ethno* or emic or etic or phenomenolog* or hermeneutic* or heidegger* or husserl* or colaizzi* or giorgi* or glaser or strauss or van kaam* or van manen or constant compar*).ti,ab.
7	Focus groups/ or Interview/ or Interviews as Topic/ or Health services administration/ or Questionnaires/ or Self-report/
8	(focus group* or grounded theory or narrative analys* or lived experience* or life experience* or theoretical sampl* or purposive sampl* or ricoeur or spiegelberg* or merleau or metasynthes* or meta-synthes* or metasummar* or meta-summar* or metastud* or meta-stud* or maximum variation or snowball).ti,ab.
9	((thematic* adj3 analys*) or (content analy* or field note* or fieldnote* or field record* or field stud*) or (participant* adj3 observ*) or (nonparticipant* adj3 observ*) or (non participant* adj3 observ*)).ti,ab.
10	(semi-structured or semistructured or structured categor* or unstructured categor* or action research or (audiorecord* or taperecord* or videorecord* or videotap*) or ((audio or tape or video*) adj5 record*) or interview* or quasi-experiment* or (case adj stud*)).ti,ab.
11	(collaborat* or consultat* or experience or involve* or narrative* or opinion* or participat* or partner* or perspective* or story or stories or "social science*" or view* or voice*).ti,ab.
12	5 or 6 or 7 or 8 or 9 or 10 or 11
13	4 and 12
