# Supplementary material for: What factors affect evidence-informed policymaking in public health? Protocol for a systematic review of qualitative evidence using thematic synthesis
Source: Syst Rev. 2016 Apr 14;5:61. doi: 10.1186/s13643-016-0240-6 (PMC4831125; doi:10.1186/s13643-016-0240-6)
Supplement: Additional file 2: — Adapted CASP form - Tool that will be used to assess the methodological quality of included studies. (PDF 27 kb) [file 13643_2016_240_MOESM2_ESM.pdf]

Assessment of study quality – Adapted CASP form

Reviewer: ________________________________ Date: __________________

Author: _______________________________ Year: _______ Record Number: _____

Assessment questions	Response (circle)		
1. Was there a clear statement of the research question(s) and/or the aim(s) of the research?	Yes	No	Unclear
2. Was a qualitative approach appropriate?	Yes	No	Unclear
3. Were the research methodology and design appropriate for addressing the research question?	Yes	No	Unclear
4. Was the sampling/recruitment strategy appropriate for addressing the research question?	Yes	No	Unclear
5. Were the methods of data collection appropriate for addressing the research question?	Yes	No	Unclear
6. Were the data analysis methods sufficiently rigorous and appropriate for addressing the research question?	Yes	No	Unclear
7. Is there a statement locating the researcher(s) culturally and/or theoretically?*	Yes	No	Unclear
8. Has the relationship between researcher and participants been adequately considered?*	Yes	No	Unclear
9. Are the participants and their voices adequately represented?*	Yes	No	Unclear
10. Have ethical issues been adequately taken into consideration, and is there evidence of ethical approval from an appropriate body?	Yes	No	Unclear
11. Is there a clear statement of findings?	Yes	No	Unclear
12. Do the conclusions drawn in the research report flow from the analysis, or interpretation, of the data?*	Yes	No	Unclear

*Item adapted from JBI-QARI qualitative critical appraisal instrument

Overall methodological rating (circle): **High** **Moderate** **Low**

Rationale and comments: ____

____

____

____

____
